# Sub-chronic inhalation of high concentrations of manganese sulfate induces lower airway pathology in rhesus monkeys

**DOI:** 10.1186/1465-9921-6-121

**Published:** 2005-10-21

**Authors:** David C Dorman, Melanie F Struve, Elizabeth A Gross, Brian A Wong, Paul C Howroyd

**Affiliations:** 1 CIIT Centers for Health Research, 6 Davis Drive, P.O. Box 12137, Research Triangle Park, NC 27709-2137, USA; 2 Experimental Pathology Laboratories, Inc., P.O. Box 12766, Research Triangle Park, NC 27709, USA

## Abstract

**Background:**

Neurotoxicity and pulmonary dysfunction are well-recognized problems associated with prolonged human exposure to high concentrations of airborne manganese. Surprisingly, histological characterization of pulmonary responses induced by manganese remains incomplete. The primary objective of this study was to characterize histologic changes in the monkey respiratory tract following manganese inhalation.

**Methods:**

Subchronic (6 hr/day, 5 days/week) inhalation exposure of young male rhesus monkeys to manganese sulfate was performed. One cohort of monkeys (n = 4–6 animals/exposure concentration) was exposed to air or manganese sulfate at 0.06, 0.3, or 1.5 mg Mn/m^3 ^for 65 exposure days. Another eight monkeys were exposed to manganese sulfate at 1.5 mg Mn/m^3 ^for 65 exposure days and held for 45 or 90 days before evaluation. A second cohort (n = 4 monkeys per time point) was exposed to manganese sulfate at 1.5 mg Mn/m^3 ^and evaluated after 15 or 33 exposure days. Evaluations included measurement of lung manganese concentrations and evaluation of respiratory histologic changes. Tissue manganese concentrations were compared for the exposure and control groups by tests for homogeneity of variance, analysis of variance, followed by Dunnett's multiple comparison. Histopathological findings were evaluated using a Pearson's Chi-Square test.

**Results:**

Animals exposed to manganese sulfate at ≥0.3 mg Mn/m^3 ^for 65 days had increased lung manganese concentrations. Exposure to manganese sulfate at 1.5 mg Mn/m^3 ^for ≥15 exposure days resulted in increased lung manganese concentrations, mild subacute bronchiolitis, alveolar duct inflammation, and proliferation of bronchus-associated lymphoid tissue. Bronchiolitis and alveolar duct inflammatory changes were absent 45 days post-exposure, suggesting that these lesions are reversible upon cessation of subchronic high-dose manganese exposure.

**Conclusion:**

High-dose subchronic manganese sulfate inhalation is associated with increased lung manganese concentrations and small airway inflammatory changes in the absence of observable clinical signs. Subchronic exposure to manganese sulfate at exposure concentrations (≤0.3 mg Mn/m^3^) similar to the current 8-hr occupational threshold limit value established for inhaled manganese was not associated with pulmonary pathology.

## Background

There is growing evidence to suggest that a wide variety of respirable particles can induce lung injury under certain exposure conditions. Clinical recognition of this hazard originally stemmed from occupational studies examining workplace exposure to metals, asbestos, silica, coal, and other biologically active particles [1,2]. However, particulate-induced lung injury is not confined to the workplace. There is strong epidemiologic evidence for significant associations between respiratory morbidity, including exacerbations of asthma and mortality, with exposure to relatively low ambient particulate matter concentrations [3,4]. This association has contributed to the adoption of more stringent ambient air quality standards for respirable particulate matter by the United States Environmental Protection Agency and other health organizations.

Particulate matter is not a single entity but rather a mixture of many subclasses of pollutants including metals, sulfate, acids, ammonium, nitrate, organic compounds, and minerals. Depending on emission source, metals may represent a significant proportion of a particulate matter sample [5]. Soluble metals have been implicated in particulate matter-associated cardiopulmonary disease in healthy and compromised individuals [6-8]. One metal found in ambient air is manganese. Airborne manganese sources include wind erosion of dusts and soils, anthropogenic fugitive dusts, and emissions from automobiles, power plants, coke ovens, municipal waste incinerators, and metal smelting operations [5]. Ambient air manganese concentrations are typically quite low, ranging between 5 and 33 ng Mn/m^3 ^[9]. Significant occupational manganese exposure (≥0.2 mg Mn/m^3^) can occur in some workers involved in ferroalloy production, iron and steel foundries, and welding [9].

Workers exposed to high atmospheric manganese concentrations frequently demonstrate an increased incidence of cough and other signs associated with bronchitis [10,11]. Acute inhalation of air with extremely high manganese concentrations (≥1 mg Mn/m^3^) can result in pneumonitis [12]. Although manganese-induced pneumonitis has been recognized since the mid-1940's, histological characterization of the pulmonary response remains incomplete. Most experimental exposures in laboratory animals have shown only minor lung pathology despite the administration of high doses of manganese oxides by intratracheal instillation or inhalation [13-18]. Far less is known about the potential respiratory effects induced by exposure to other inorganic forms of manganese. Manganese chloride instillation into rabbits failed to induce significant pulmonary pathology [19]. Dorman et al. [20] showed that subchronic exposure of rats to manganese sulfate (MnSO_4_) was associated with rhinitis in the anterior part of the nose. To our knowledge, histologic assessment of pulmonary changes occurring in association with subchronic inhalation of MnSO_4 _has not been performed and is the subject of our study. Herein, we report that subchronic high-dose inhalation exposure of nonhuman primates to MnSO_4 _resulted in mild subacute bronchiolitis, alveolar duct inflammation, and proliferation of bronchus-associated lymphoid tissue (BALT), in the absence of rhinitis or other forms of nasal pathology.

## Methods

### Chemicals

Manganese (II) sulfate monohydrate (MnSO_4_·H_2_O) was obtained from Aldrich Chemical Company, Inc. (Milwaukee, WI).

### Animals

This study was conducted under federal guidelines for the care and use of laboratory animals [21] and was approved by the CIIT Centers for Health Research (CIIT) Institutional Animal Care and Use Committee. Additional endpoints evaluated in this study, but not presented in the present manuscript, included determination of manganese concentrations in additional tissues and magnetic resonance imaging (MRI) of the brain. We chose to use rhesus monkeys since they are extensively used in toxicology studies, manganese-exposed monkeys develop distribution patterns for this metal within the brain that mimic those seen in heavily exposed people [22], and there are anatomically-based simulation models for air flow in the macaque upper and lower respiratory tracts [23,24]. Thirty-six male rhesus monkeys purchased from Covance Research Products, Inc. (Alice, TX) were used in this study. Monkeys were 17 to 22 months old at the time of their arrival at CIIT. Animals were screened for the nasal parasite *Anatrichosoma spp*. herpes B, simian immunodeficiency virus, simian respiratory virus, pulmonary acariasis, and tuberculosis, and were subjected to a thorough clinical examination including an evaluation of pre-exposure blood samples for routine hematology and clinical chemistry. The results of these evaluations were within normal limits.

Assignment of animals to treatment cohorts considered the age of the animal. Assignment occurred so that the animals were between 20 and 24 months of age at the start of the inhalation exposure. Randomization of animals to treatment groups occurred prior to the start of the inhalation exposure and was based upon a weight randomization procedure. Animals were acclimated to the facility for at least 43 days prior to the start of the first inhalation exposure. Additional endpoints evaluated in this study, but not presented in the present manuscript, included magnetic resonance imaging (MRI) of the brain, post-exposure clinical chemistry and hematological evaluations, and determination of tissue manganese concentrations in the central nervous system and other organs.

### Animal Husbandry

Animal rooms were maintained at daily temperatures of 22 ± 4°C, relative humidity of 30–70%, and an air flow rate sufficient to provide 10–15 air changes per hour. Lighting was controlled by automatic controls (lights on approximately 0600–1800). All exposures were conducted during the animal's light cycle (approximately from 0800 to 1400). All animals were housed in animal rooms or exposure chambers within CIIT's animal facility. This facility is accredited by the Association for Assessment and Accreditation of Laboratory Animal Care, International. A certified primate chow (# 5048) diet from Purina Mills (St. Louis, MO) was fed twice-a-day (total daily amount fed was approximately 4% of the animal's body weight). Dietary supplements were also used as part of CIIT's nonhuman primate enrichment program. These supplements included fruits (e.g., oranges, raisins, apples), vegetables (e.g., carrots), and treats (e.g., honey, candies, cereal, fruit juices) purchased from a local grocery store. Reverse osmosis purified water was available *ad libitum*. During non-exposure periods, domiciliary stainless steel cages (0.4 m^2 ^× 0.8 m tall) suitable for housing macaque monkeys (Lab Products, Inc.; Seaford, DE) were used to individually house monkeys. On each exposure day, animals were transferred to 0.2 m^2 ^× 0.6 m tall stainless steel cages (Lab Products, Inc.; Seaford, DE) that were designed to fit within the 8-m^3 ^inhalation chambers. Animals were moved back to their domiciliary cages after the end of each 6-hr exposure.

### Manganese Exposures

**Figure 1:**
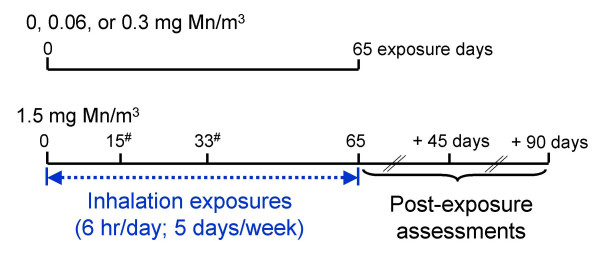
**Experimental design overview**. Group size equals 4 monkeys per exposure group, with the exception of the 0 and 0.06 mg Mn/m^3 ^exposure groups (n = 6 monkeys/exposure concentration). Lung manganese concentrations and respiratory histologic changes were evaluated after 15, 33, or 65 exposure days or 45 or 90 days after the 65^th ^exposure day. ^#^Denotes animals assigned to cohort 2.

MnSO_4 _aerosol concentrations of 0.18, 0.92, and 4.62 mg MnSO_4_/m^3^, corresponding to 0.06, 0.3, and 1.5 mg Mn/m^3^, were generated for this study. Control animals were exposed to filtered air. Animal exposures were conducted as described in Figure 1. Four 8-m^3 ^stainless steel and glass inhalation exposure chambers with glass doors and windows for animal observation were used. Animal position within the inhalation chambers was rotated weekly to minimize the impact of any undetected differences in the environment or in the MnSO_4 _exposure concentrations. Air flow through the 8-m^3 ^inhalation chamber was typically maintained at a rate sufficient to provide at least 12 air changes per hour. Airflow through each chamber was monitored continuously during each exposure and recorded every 30 minutes. Temperature and relative humidity inside each inhalation chamber were recorded every 30 minutes during each 6-hr exposure. The average chamber temperature and relative humidity during the 6-hr exposure period were maintained at 18–26°C and 50 ± 20%, respectively. Methods describing the generation of the MnSO_4 _atmosphere with a dry powder generator (Wright Dust Feeder, Model WDF-II, BGI, Inc., Waltham, MA) and the characterization of the subsequent aerosol have been previously described [25]. MnSO_4 _was packed in separate dry powder generator cups at pressures between 2000 and 3000 psi (Model 3912, Carver Inc., Wabash, IN) using a hydraulic press (Model C, Carver Inc., Menomonee Falls, WI).

### Necropsy Procedures

Necropsies were performed the day following the last inhalation exposure (i.e., 12–18 hr after termination of the final inhalation exposure). Food was withheld overnight prior to necropsy. Monkeys were anesthetized with ketamine (20 mg/kg, IM, Fort Dodge Animal Health, Fort Dodge, IA) and euthanized with pentobarbital (80–150 mg/kg, IV, Henry Schein Inc., Port Washington, NY) followed by exsanguination. Following euthanasia, the lungs and other thoracic organs were removed, weighed, and inspected for gross lesions. The left primary bronchus was ligated and the left lung separated for determination of tissue manganese concentration. The right lung and trachea were then inflated with 10% neutral-buffered formalin using 30-cm of hydrostatic pressure [26]. The olfactory epithelium was excised for chemical analysis and the remaining nasal tissues, including the nasopharynx and larynx, were stored in 10% neutral-buffered formalin. The larynx samples were decalcified in 10% formic acid (Fisher Scientific International, Inc., Hampton, NH) for two days. Cranial tissues were decalcified in RDO^® ^(Apex Engineering Products Corporation, Plainfield, IL) for up to 6 days. Following decalcification, tissues were washed in running tap water for at least 6 hr. Following fixation (and decalcification when appropriate), representative samples of the lung, trachea, larynx, oropharynx, tracheobronchial lymph nodes, and nose were collected from each animal. Tissue samples were trimmed, embedded in paraffin, and five-μm thick sections were cut and stained with hematoxylin and eosin for light microscopic evaluation. Histologic specimens were examined by an experienced veterinary pathologist (Howroyd).

### Tissue manganese concentrations

Tissue samples collected for chemical analyses were stored in individual plastic containers, frozen in liquid nitrogen, and stored at approximately -80°C until chemical analyses were performed. Lung and olfactory epithelium manganese concentrations were determined by graphite furnace atomic absorption spectrometry using previously published methods [27].

### Statistics

Tissue manganese concentrations were compared for the exposure and control groups by tests for homogeneity of variance (Levene's test), analysis of variance (ANOVA), and Dunnett's multiple comparison procedure for significant ANOVA. Histopathological findings were evaluated using a Chi-Square test. Statistical analyses were performed using SAS Statistical Software. A probability value of <0.01 was used for Levene's test, while <0.05 was used as the critical level of significance for all other statistical tests. Unless otherwise noted, data presented are mean values ± standard error of the mean (SEM).

## Results

### Test atmospheres

No significant differences in the test aerosol characteristics were observed between the two exposure cohorts (Table 1). Particles of unknown composition (arising from animal dander and other background sources) were present in the control chamber at an overall average concentration ± standard deviation (SD) of 0.004 ± 0.002 mg/m^3^. The calculated mass median aerodynamic diameter (MMAD) for the particles in the control chamber was 3.9 μm.

**Table 1:** Characteristics of manganese aerosols generated for whole-body exposures in this study (means ± SD)

	Nominal MnSO_4 _exposure concentration (mg/m^3^)			
	0.18	0.92	4.62^a^	4.62^b^
Actual exposure concentration (mg MnSO_4_/m^3^)^c^	0.19 ± 0.01	0.97 ± 0.06	4.55 ± 0.33	4.45 ± 0.35
Geometric mean diameter (μm)^d^	1.04	1.07	1.12	1.04
Geometric standard deviation (σ_g_)^d^	1.51	1.54	1.58	1.50
Mass median aerodynamic diameter (μm)^e^	1.73	1.89	2.12	1.72

^a ^Cohort 1

^b ^Cohort 2

^c ^Based on continuous chamber monitoring with a calibrated optical particle sensor (Real-Time Aerosol Sensors, Model RAM-S, MIE, Inc., Billerica, MA).

^d ^Based on biweekly aerodynamic particle size spectrometry (Aerodynamic Particle Sizer, Model 3320, TSI, Inc., St. Paul, MN) measurements.

^e ^Calculated value [56]

### Lung and olfactory epithelium manganese concentrations following MnSO_4 _exposure

Lung and olfactory epithelium manganese concentrations are presented in Table 2. Animals exposed to MnSO_4 _at ≥0.3 mg Mn/m^3 ^for 65 exposure days developed increased lung manganese concentrations. Animals exposed to MnSO_4 _at ≥0.06 mg Mn/m^3 ^for 65 exposure days developed increased olfactory epithelium manganese concentrations. Increased lung and olfactory epithelium manganese concentrations developed within three weeks of exposure to MnSO_4 _at 1.5 mg Mn/m^3^. Within 45 days after completion of the 65-day inhalation exposure to MnSO_4 _at 1.5 mg Mn/m^3 ^regimen, lung and olfactory epithelium manganese concentrations were not different from those seen in air-exposed controls.

**Table 2:** Olfactory epithelial and lung manganese concentrations in young monkeys following exposure to air or MnSO_4_. Manganese concentrations were determined by graphite furnace atomic absorption spectrometry and are expressed as mean ± SEM μg Mn/g tissue wet weight. Young male rhesus monkeys (n = 4 except where noted) were exposed to either air or MnSO_4 _6 hours/day, 5 days/week.

Tissue	MnSO_4 _exposure concentration (mg Mn/m^3^)	15	33	Exposure Day 65	65 [+45]^a^	65 [+90]^a^
Olfactory epithelium	0			0.42 ± 0.01^b^		
	0.06			1.22 ± 0.15*^b^		
	0.3			2.96 ± 0.46*		
	1.5	6.10 ± 0.39*	7.34 ± 0.70*	7.10 ± 2.01*	0.65 ± 0.04	0.69 ± 0.11

Lung	0			0.15 ± 0.03^b^		
	0.06			0.18 ± 0.01^b^		
	0.3			0.25 ± 0.02*		
	1.5	0.39 ± 0.06*	0.35 ± 0.02*	0.33 ± 0.04*	0.09 ± 0.01	0.06 ± 0.01

^a ^Number in brackets indicates number of days post exposure assessment.

^b ^n = 6.

* p < 0.05

### Respiratory tract pathology following MnSO_4 _exposure

**Figure 2:**
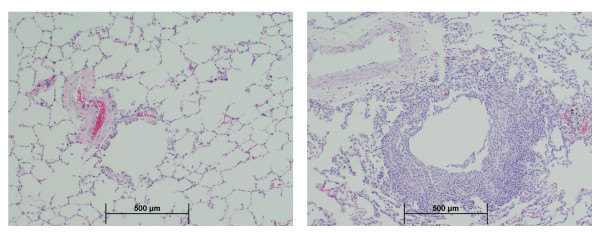
**Bronchiolitis**. Moderate subacute bronchiolitis in a monkey exposed to the highest (1.5 mg Mn/m^3^) MnSO_4 _exposure concentration for 15 exposure days (right). Normal appearing bronchioles present in an air-exposed control monkey (left). (10×)

**Figure 3:**
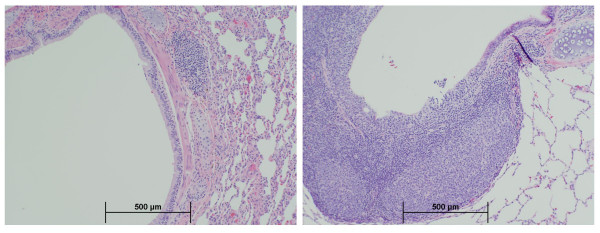
**BALT proliferation**. Peribronchial BALT proliferation in a monkey exposed to the highest (1.5 mg Mn/m^3^) MnSO_4 _exposure concentration for 65 exposure days (right). Normal appearing BALT present in an air-exposed control monkey (left). (4×)

Manganese exposure did not affect absolute or relative lung weights and did not result in coughing, dyspnea, or other respiratory signs (data not shown). High-dose exposure to MnSO_4 _was associated with an increased incidence of minimal to mild subacute bronchiolitis (Table 3). These lesions consisted of infiltrates of lymphocytes, along with neutrophils and occasional eosinophils, primarily surrounding the terminal and respiratory bronchioles and/or alveolar ducts, but sometimes extending into the lamina propria (Figure 2). Macrophages with moderate amounts of pale-staining cytoplasm were occasionally observed in the adjacent airway lumen. Although the overlying epithelium generally appeared intact, because it is normally very thin at the level of the distal airways, it is difficult to confirm that epithelial integrity was unaffected. These changes appeared to be reversible upon cessation of MnSO_4 _exposure. An increased incidence of enhanced proliferation of BALT in association with smaller (≤500 μm) airways also occurred in monkeys exposed to the highest MnSO_4 _concentration (1.5 mg Mn/m^3^) (Figure 3). Some BALT foci included germinal center formation. The incidence of increased BALT was highest in monkeys exposed to 1.5 mg Mn/m^3^for 33 exposure days (Table 3) suggesting that BALT proliferation may subside even in the face of ongoing manganese exposure. Proliferation of BALT occurred in one animal exposed to MnSO_4 _at 0.3 mg Mn/m^3^; however, this increase was only minimal and was not statistically significant.

**Table 3:** Incidence of MnSO_4_-induced microscopic lesions observed in young male rhesus monkeys exposed to MnSO_4_. Incidence is expressed as number affected/number examined.

Lesion	MnSO_4 _exposure concentration (mg Mn/m^3^)	15	33	Exposure Day 65	65 [+45]^a^	65 [+90]^a^
Subacute bronchiolitis/alveolar duct inflammation	0			0/6		
	0.06			0/6		
	0.3			1/4^b^		
	1.5	3/4*	4/4*	3/4*	0/4	1/4

Increased bronchus associated lymphoid tissue	0			0/6		
	0.06			0/6		
	0.3			1/4		
	1.5	2/4^†^	3/4*	1/4	2/4^†^	1/4

^a ^Number in brackets indicates number of days post exposure assessment

^b ^Subacute bronchiolitis was observed in one monkey; however, in this animal only, this lesion was observed in conjunction with aspirated food particles.

* p < 0.05 (Pearson's chi-square test)

^† ^p = 0.053 (Pearson's chi-square test)

Several of the MnSO_4_-exposed animals had minimal acute alveolitis. However, this finding was considered unlikely to be treatment-related since no statistically significant dose-response relationship was observed. Minimal changes consistent with chronic bronchiolitis were observed in several control as well as MnSO_4_-exposed animals. As such, these changes were not considered to be treatment-related. The majority of lung sections examined, including those taken from controls, had scattered deposits of brown-green pigment in the interstitial tissue. Such chronic bronchiolitic changes characterized by lung pigment deposits are commonly seen in monkeys with lung mites [28,29]. The most common lung mite is *Pneumonyssus simicola *and this agent occurs with nearly 100% incidence in rhesus monkeys [28,29]. Animals used on this study were treated with ivermectin prior to use; thus it is unlikely that superimposed active mite infection occurred. This conclusion is further supported by the absence of microscopic evidence of mites.

**Figure 4:**
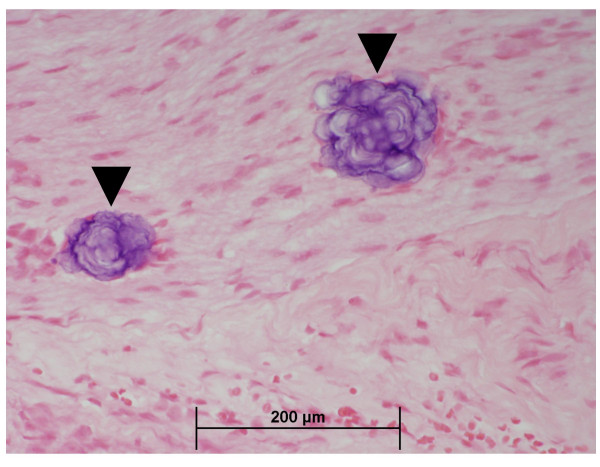
**Basophilic foci in nerves**. Basophilic foci (arrow) in nerves of olfactory mucosa from a monkey exposed to MnSO_4 _at 0.06 mg Mn/m^3^, for 65 days (40×).

One animal from each of the 0.06 and 1.5 mg Mn/m^3 ^exposure groups had minimal or mild, basophilic foci in the nerves of the nose. The spherical basophilic foci were approximately 40 μm in diameter and consisted of concentric lamellae (Figure 4). The foci were Periodic acid-Schiff stain positive but failed to stain with alizarin red, von Kossa's, or Perl's stains (for calcium or iron) and were not birefringent in polarized light (data not shown). Similar foci were present in the nasal epithelium of three other animals, including one control animal. Thus, it is unlikely that these foci were induced by MnSO_4 _exposure. X-ray microanalysis of foci taken from the decalcified nasal epithelium of a single monkey from the 1.5 mg Mn/m^3 ^exposure group showed the presence of sulfur but no other ions (data not shown). Thus, the basophilic foci noted in the nasal nerves were most likely glycoproteinaceous inclusion bodies (i.e., corpora amylacea) or psammoma bodies. Similar deposits have been observed in the olfactory nerves of untreated rhesus monkeys and rats (Howroyd, unpublished observations), in the olfactory tracts of humans [30], and in the brains of mice [31] and monkeys [32,33].

## Discussion

Pulmonary inflammation is a common response to the inhalation of various types of particles including manganese [34]. In the present study, monkeys exposed to the highest MnSO_4 _concentration (1.5 mg Mn/m^3^) developed subacute bronchiolitis and proliferation of BALT. Bronchiolitis included an infiltrate of acute inflammatory cells (neutrophils) in the peribronchiolar connective tissues in the centriacinar region. These lesions developed relatively rapidly as they were observed in monkeys exposed to MnSO_4 _for only 15 exposure days. However, with ongoing MnSO_4 _exposure, the bronchiolitic lesion progressed, affecting a larger percentage of respiratory bronchioles examined. Bronchiolitis resolved rapidly after cessation of MnSO_4 _exposure and was absent 45 days after the end of the 13 week exposure to MnSO_4 _at 1.5 mg Mn/m^3^.

Monkeys and human beings share similar lung anatomy [35], and their respiratory bronchioles and other small airways have similar sensitivity to inhaled toxicants such as cigarette smoke, coal dust, and ozone [36-38].

Following bronchiolitis, people are often noted to have increased wheeze, cough, and asthma, increased airway responsiveness, and reduced lung function due primarily to airflow obstruction [39]. In the present study, signs referable to the respiratory system were not recognized in the monkeys developing manganese-induced bronchiolitis. Although pulmonary function was not assessed in our study, reduced forced expiratory volume in one second (FEV_1) _and forced vital capacity (FVC) have been reported in workers that have been chronically exposed to high levels of manganese dust [10,40].

Particles deposited in the lung can be retained in the lung interstitium [41] or cleared via the mucociliary apparatus or through the lymphatic system [42]. Alternatively, particles may be transported from the lung via alveolar macrophages or neutrophils with subsequent accumulation in the BALT and tracheobronchial lymph node [42,43]. Thus, as a lymphoepithelial organ, the BALT is critical to the immune defense of the lung and to alveolar clearance of particles and lung pathogens. Proliferation of BALT has been observed in rodents following inhalation exposure to carbon black and silica [44,45]. There is evidence that BALT proliferation occurs in humans with panbronchiolitis, chronic hypersensitivity pneumonitis, and other chronic inflammatory airway disease [46,47]. Proliferative BALT lesions observed in these disease conditions are comparable to those observed in the MnSO_4_-exposed monkeys, suggesting that BALT proliferation in the manganese-exposed monkeys may be related to the local airway immune response secondary to inflammation induced by MnSO_4_.

Some agents that target the lung may also affect the upper airways as well. Our laboratory has recently reported that rats exposed subchronically to MnSO_4 _(at 0.5 mg Mn/m^3^) develop a mild reversible inflammatory change consisting of pleocellular inflammatory infiltrates and fibrinonecrotic debris within the nasal respiratory epithelium [20]. These lesions occurred primarily in high airflow regions and were consistent with mild irritation. In the present study, however, despite a 15-fold increase in nasal epithelial manganese concentrations, we did not observe any chemical-related nasal pathology in monkeys exposed to MnSO_4_. Although nasal pathology did not occur, the fate of the manganese that is initially deposited in the nose and subsequently absorbed by the olfactory epithelium remains toxicologically important. Experiments from our laboratory have shown that manganese deposited on the olfactory epithelium can undergo transport along the olfactory nerve with subsequent delivery to the olfactory bulb [48].

Our interest in MnSO_4 _stems from the use of manganese in the gasoline fuel additive methylcyclopentadienyl manganese tricarbonyl (MMT). Automobiles that use MMT in the fuel and are equipped with catalytic converters emit manganese primarily in the phosphate and sulfate forms with smaller amounts of manganese oxides also being discharged [49]. The manganese exposure concentrations used in this study bracket several human exposure scenarios. Prolonged human exposure to the highest MnSO_4 _concentration used in this study (1.5 mg Mn/m^3^) can occur among manganese miners and prolonged exposure is associated with frank neurotoxicity and respiratory disease [9]. Our mid-dose exposure concentration is analogous to the current 8-hr Threshold Limit Value (TLV) for inhaled manganese of 0.2 mg Mn/m^3 ^that has been established by the American Conference of Governmental Industrial Hygienists (ACGIH). Our lowest exposure concentration (0.06 mg Mn/m^3^) is > 2,000-fold higher than typical air manganese concentrations observed in the ambient air including Canadian cities where MMT is extensively used in gasoline [9,50]. In the present study, exposure concentrations associated with increased lung manganese concentrations and lung pathology were respectively 1.5-and 7.5-fold higher than the current TLV.

The MnSO_4 _particle size used in this study had an MMAD of 1.72 to 2.12 μm. Aerodynamic size is an important factor that influences particle deposition [51]. Several models based on airway geometry have been developed to describe particle deposition patterns in humans, monkeys, and rats [52,53]. The model of Asgharian and coworkers (1995) predicts a pulmonary deposition efficiency of an aerosol with a particle size of 1.5 μm of approximately 35% for humans and rhesus monkeys while the rat had much lower deposition efficiency (6%) due to higher nasal uptake [52]. The rate of particle clearance from the alveolar region also differs among species. Rodents clear particles from the lung more quickly than either monkeys or humans [54]. Anatomical differences between rodents and primates also affect particle deposition, retention, and clearance. Rodents lack respiratory bronchioles and have simple acini. Macaque monkeys and humans have larger alveoli and alveolar ducts than rats [55] and have similar numbers of respiratory bronchiole generations between the terminal bronchiole and the alveolar duct [35,56]. Our results may be especially important for human risk assessment owing to the fact that monkeys and human beings share similar lung anatomy (i.e., both species have extensive respiratory bronchioles comprised of bronchiolar epithelium and gas exchange epithelium) [35].

## Conclusion

High-dose subchronic manganese sulfate inhalation is associated with increased lung manganese concentrations, mild subacute bronchiolitis, alveolar duct inflammation, and proliferation of bronchus-associated lymphoid tissue. Bronchiolitis and alveolar duct inflammatory changes were absent 45 days post-exposure, suggesting that these lesions are reversible upon cessation of subchronic high-dose manganese exposure. These small airway changes occurred in the absence of observable clinical signs. Subchronic exposure to manganese sulfate at exposure concentrations (≤0.3 mg Mn/m^3^) similar to the current 8-hr occupational threshold limit value established for inhaled manganese was not associated with pulmonary or nasal pathology.

## Competing interests

This publication is based on a study sponsored and funded by Afton Chemical Corporation in satisfaction of registration requirements arising under Section 211(a) and (b) of the Clean Air Act and corresponding regulations at 40 C. F. R. Subsections 79.50 *et seq*.

## Authors' contributions

DCD conceived of the study, was the principal investigator and participated in all phases of the study, and drafted the manuscript. MFS participated in the design, coordination, and conduct of the study. EAG participated in and supervised the necropsy and preparation of histology specimens. BAW designed the exposure generation system and characterization of the aerosol. PCH was the study pathologist and conducted the histopathological evaluation of tissues. All authors contributed to, read, and approved the final manuscript.
